# Critical Issues for Patients and Caregivers in Neuro-Oncology during the COVID-19 Pandemic: What We Have Learnt from an Observational Study

**DOI:** 10.3390/curroncol31070288

**Published:** 2024-07-04

**Authors:** Elena Anghileri, Irene Tramacere, Sara Morlino, Catia Leuzzi, Lorena Pareja Gutierrez, Saba Motta, Antonio Silvani, Anna Amato, Francesca Romana Berrini

**Affiliations:** 1 Neuro-Oncology Unit, Fondazione IRCCS Istituto Neurologico Carlo Besta, Via Celoria 11, 20133 Milan, Italy; antonio.silvani@istituto-besta.it; 2 Department of Research and Clinical Development, Fondazione IRCCS Istituto Neurologico Carlo Besta, 20133 Milan, Italy; tramacere.irene@gmail.com; 3 Radiotherapy Unit, Neurosurgery Department, Fondazione IRCCS Istituto Neurologico Carlo Besta, 20133 Milan, Italy; sara.morlino@istituto-besta.it; 4 Clinical Neuro-Science Department, Fondazione IRCCS Istituto Neurologico Carlo Besta, 20133 Milan, Italy; catia.leuzzi@istituto-besta.it (C.L.); lorena.gutierrez@istituto-besta.it (L.P.G.); 5 Scientific and Patients Library, Fondazione IRCCS Istituto Neurologico Carlo Besta, 20133 Milan, Italy; saba.motta@istituto-besta.it; 6 Department of Medicine and Surgery, University of Milano-Bicocca, 20126 Milan, Italy; a.amato29@campus.unimib.it; 7 AITC-Associazione Italiana Tumori Cerebrali (Italian Brain Tumor Association), 20133 Milan, Italy; berrinifrancesca@gmail.com

**Keywords:** anxiety, caregivers, caregiver burden, communication, COVID-19, (brain) neoplasms, pandemics, perception, psycho-oncology, quality of life

## Abstract

Objective: The COVID-19 pandemic affected neuro-oncological patients and their caregivers regarding tumor care and emotional functioning, including Quality of Life (QoL). This study aimed to understand how COVID-19 affected their psychological state and the relations between patients and health personnel in neuro-oncology. Methods: A cross-sectional study was conducted on neuro-oncological patients and their caregivers. Results: A total of 162 patients and 66 caregivers completed the questionnaire. Altogether, 37.5% of patients perceived a greater risk of contracting COVID-19 compared to the general population. On a 0–10 scale, the patients’ tumor-related anxiety score was 5.8, and their COVID-19-related score was 4.6. The caregivers reported 7.7 and 5.5, respectively. QoL was described as at least good in 75% of both patients and caregivers; the caregivers’ care burden increased in 22.7% of cases during the pandemic, with no correlation with QoL. Future perception often changed, both in patients and caregivers. In 18% of cases, the cancer treatment schedule was changed, either by patient decision or by medical decision. However, 93.5% of patients were satisfied with their overall care. Conclusions: A considerable proportion of patients and caregivers still perceived the tumor disease as more burdensome than the pandemic, and their future as more uncertain. Such data suggest the need to build a productive alliance between patients and health professionals.

## 1. Introduction

Historically, the concept of “health” has included both body care and mind care: the World Health Organization (WHO) (1948) defines health as “a state of complete physical, mental, and social well-being and not merely the absence of disease or infirmity”, referring to the biopsychosocial model. It is a broad concept that is influenced in complex ways by physical health, psychological state, level of independence, social relationships, and connections to salient features of the environment. Ideal quality of life is a state of complete physical, mental, and social well-being [1]. In the context of disease, reference is usually made to a specific aspect of quality of life, health-related (HR) quality of life (HR QoL), referring to an individual’s perception of their position in life in the context of culture and value systems. QoL is influenced not only by symptoms of the disease or side effects of treatment, but also by psychological and socioeconomic factors [2].

Cancer diagnosis as well as COVID-19 risk or disease are described as contexts of high fragility. Frailty describes a state of increased vulnerability and decreased physiological reserve that can be defined by multidimensional components, including physical, psychological, and social factors. Several theories have been developed over the decades (https://frailtyscience.org/conceptual-models-frailty/, accessedon 15 June 2024), including Lipsitz’s Loss of Complexity [3] and Varadhan’s Dynamical System Theory [4]. They report that frailty develops when the complex feedback loops that maintain homeostasis lose their adaptability, leading to larger perturbations in function that ultimately manifest as dysfunction. We address this topic with respect to the population with a diagnosis of brain cancer during the COVID-19 pandemic. A cancer diagnosis inherently leads to various existential fears [5], and, in a similar setting, so also did the COVID-19 pandemic. Besides addressing how the disease’s psychological burden was influenced by the COVID-19 pandemic, we also focused on the level of resilience and possible vigor that can develop as a positive reaction [6].

In this context, the role of healthcare professionals is to ensure and promote better standards of care, which is also based on the real needs of patients and caregivers and on the historical and social setting, especially in a pandemic period such as that of COVID-19, where functional status may be compromised. In fact, good medical practice cannot be separated from the satisfaction of patients’ and their caregivers’ psychological needs.

Caregivers are mostly informal, such as relatives and friends, and they help people with cancer during and after treatment. Specifically, they help with daily needs, doing or arranging housework, managing finances, planning for care and services, visiting often and providing emotional support. Less frequently, patients are supported by formal caregivers that are trained professionals and are paid to provide care for patients [https://www.cancer.org/cancer/caregivers/what-a-caregiver-does/who-and-what-are-caregivers.html, accessed on 15 June 2024]. Approximately 7% of the US population is made up of family caregivers for loved ones with cancer, and 4% of the US population is surviving cancer, meaning the ratio of family caregivers to cancer survivors is nearly double, supporting the notion that cancer not only affects the individual diagnosed but rather impacts an entire family unit and network of close friends [https://www.cancer.org/research/cancer-facts-statistics/all-cancer-facts-figures/cancer-facts-figures-2018.html, accessed on 15 June 2024].

Since the COVID-19 pandemic began, there has been considerable emphasis placed on the implications for patients with cancer in terms of their vulnerability to SARS-CoV-2 virus in healthcare settings. There is also concern that cancer patients and cancer survivors are more likely to get infected with the novel coronavirus and are more likely to die from complications of COVID-19 [7].

Among other cancers, neuro-oncological patients are often characterized by a dismal prognosis: they deal daily with impaired functioning, a sensation of uncertainty, fear, and difficult medical decisions. Potential neurological deficits lead to a unique symptom profile, considerably impairing the patients themselves and hindering their caregivers in everyday life. Patients can experience physical limitations, neurocognitive deficits or speech disorders, resulting in social isolation as well as an interruption to work or studying or a career break. Such a context of isolation is more severe in neuro-oncological patients compared to oncological ones due to their specific clinical impairment.

In addition to physical decline and increasing social isolation, patients may undergo a shattering of preconscious assumptions about their life and its meaning, causing existential anxiety. Then, the development of adaptive strategies to deal with the disease burden is mandatory, both for patients and caregivers. Coping strategies are a determinant factor in the process of emotional adaptation to the disease and may change over disease evolution; they are influenced by several variables, such as QoL, cognitive function, different psychological distress features, clinical condition, and disease awareness [8,9]. In addition, such strategies exhibit an important role in the dynamic interplay between the dyad made by the patient and his/her main caregiver [10].

In addition, but limited to a few studies, the emotional and social impact on the patients and their caregivers as the relationship between doctor and patients changes, including already complex and delicate communication modalities, has been addressed [11,12,13]. In parallel, clinicians have also reported the need to understand patients’ unique experiences, to communicate sensitively and empathically with patients and their caregivers as to what to expect, and to plan timely and appropriate interventions within a dynamic real-time perspective from before diagnosis to exitus [8]. During the pandemic, the main reasons for possible relationship modifications between patients/caregivers and doctors were related to the restrictions on medical facilities, such as reduced non-emergency hospitalization and reduced access to physicians; also, due to patients being reluctant to attend in person because of the fear of interacting with others, they were sometimes limited to the use of video or teleconsultations, when these options were offered [11,12,13].

Patients with malignancies have been described as experiencing higher rates of distress, anxiety, and depression than the general population, and the slower the course of treatment, the higher the distress would be [11,12,13]. The emotional state of patients and their perspectives and experiences related to the disease should be not neglected in order to promote compliance with treatments. Assessment of the needs and perceptions of patients and their caregivers appears to be a priority in order to ensure an adequate standard of care, especially in a context of collective difficulty such as a pandemic. In addition, patients with primary brain tumors mostly have a poor prognosis, although the best standard of care is followed.

The main aim of this study is to gain an extensive understanding of the impact on *mental* health and well-being of the COVID-19 pandemic in neuro-oncological patients and their caregivers. In particular, its specific aims are as follows: (I) to determine how COVID-19 affects neuro-oncological patients’ and their caregivers’ emotional state and future perception; (II) to explore patients’ and their caregivers’ needs; (III) to ascertain the relationship between patients and medical institutions, as well as patients’ compliance with care, including therapies.

## 2. Material and Methods

Neo-CO protocol is a mono-institutional observational study. Informed consent was obtained from all patients and caregivers. This study was approved by the medical ethics review board of REGIONE LOMBARDIA—SEZIONE FONDAZIONE IRCCS ISTITUTO NEUROLOGICO CARLO BESTA (minute number 72, 6th May 2020).

Inclusion criteria were as follows: subjects with a primary brain tumor, over 18 years of age; the caregiver of the patient diagnosed with a brain tumor, if present; participants able and willing to provide written informed consent and comply with the study protocol. An exclusion criterion was the inability of the subject to understand the purpose of the study.

A 41-question survey and a 16-question survey were submitted to the patients diagnosed with brain neoplasia and their caregivers, respectively (see Supplementary Materials S1 and S2). The study was proposed to the patients and caregivers in outpatient as well as inpatient services, and participation was completely voluntary. Data were collected from patients and caregiver relatives seen at the neuro-oncology department of the Fondazione IRCCS Istituto Neurologico Carlo Besta from April 2020 to December 2021. The staff of the Neuro-Oncology Information Point also contributed to the submission of the paper questionnaire, its subsequent reformulation in a digital version, and the development of information material for its dissemination and compilation online, which participants could access through dedicated links and QR codes.

The primary research question of the present study was to explore the impact of the COVID-19 pandemic on neuro-oncological patients and their caregivers. The survey topics included demographic data, COVID-19 in the surrounding area, unease and support/management related to COVID-19 and the neuro-oncology disease (such as QoL), everyday life, quality of information about the COVID-19 pandemic, and future perception.

### Statistical Analysis

Descriptive statistics were provided in terms of absolute numbers and percentages for categorical data, and means with standard deviations (SDs). Associations between variables were investigated through Fisher’s exact tests and Spearman correlation coefficients with the corresponding *p*-values, as appropriate.

## 3. Results

### 3.1. Population

From 250 patients and 150 caregivers screened, a total of 162 patients and 66 caregivers over 18 years-old completed the questionnaire.

The demographic features are reported in Table 1.

The most common diagnoses were gliomas (46.05%) and meningiomas (25.00%); less common were ependymoma (5.26%), medulloblastoma (6.58%), Primary Central Nervous System Lymphoma (PCNSL) (1.32%), neurinoma (3.29%), and other (12.50%). Disease duration was <3 months in 6.17%, 3–6 months in 14.20%, 6–12 months in 9.26%, 1–5 years in 29.01% and >5 years in 41.36% of cases.

Altogether, 9.26% of patients caught the SARS-CoV-2 infection.

For caregivers’ demographic details, see Table 1 (sex of patients, sex of caregivers, age of caregivers, nationality of caregivers, kinship of patients/caregivers).

The patients obtained information about COVID-19 from different sources, as shown in Table 1C. Most of them (77.2%) were satisfied with the information provided.

### 3.2. Perceptions of Patients and Their Caregivers: Emotional State and Future Perception

Of the patients, 37.50% perceived a greater risk of contracting COVID-19 compared to the general population, while 57.50% perceived the same risk and 5.00% a lower risk than the general population. 

**Figure 1:**
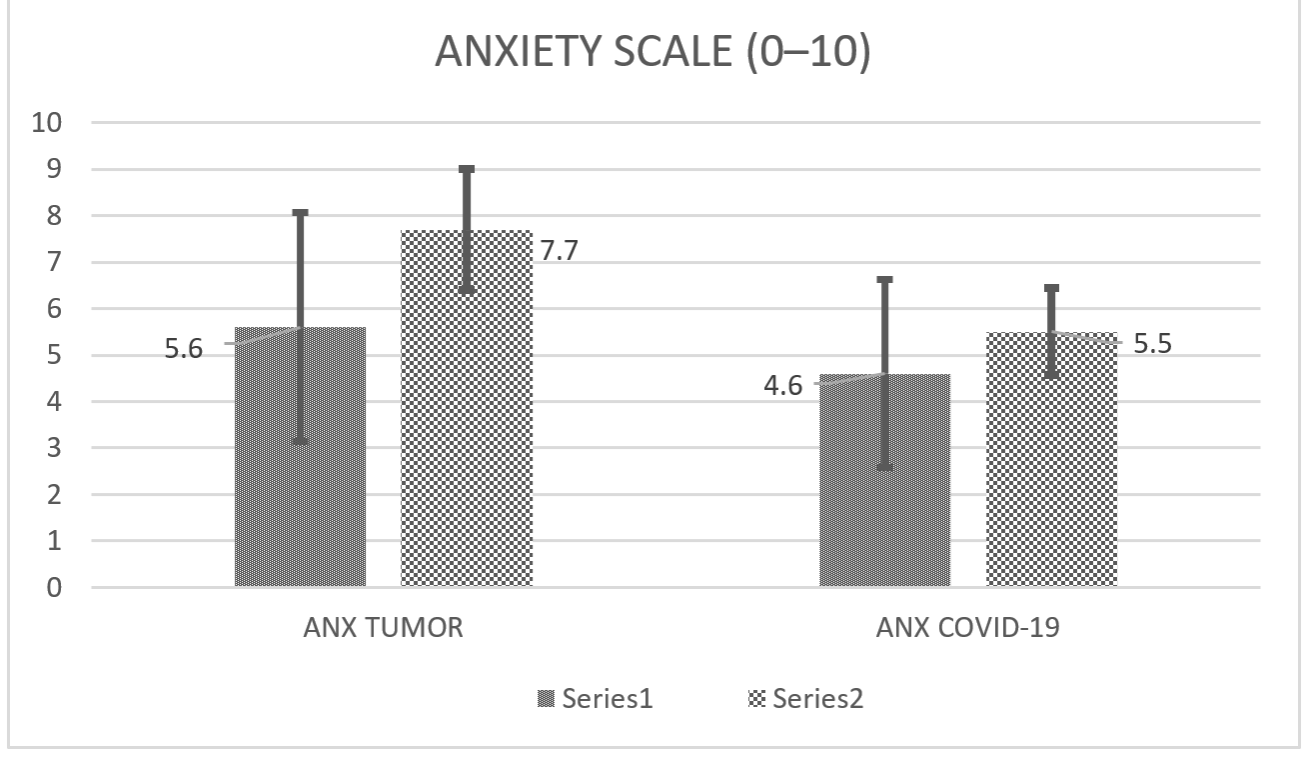
Anxiety scale. Columns show the anxiety level (range 0–10) reported by patients (small dotted) and caregivers (large dotted) for the tumor and COVID-19 pandemic, respectively. The exact value is reported at the side of the column.

Using a scale of 0–10 for the assessment of anxiety, patients experienced a 5.8 (standard deviation, SD 2.5) anxiety level related to the tumor and a 4.6 (SD 2.2) level related to COVID-19 risk (Figure 1). Caregivers experienced a 7.7 (SD 2.3) anxiety level about the tumor and a 5.5 (SD 2.2) level about COVID-19 risk (Figure 1). No significant correlations were found between patients’ and caregivers’ anxiety concerning the tumor or COVID-19.

**Figure 2:**
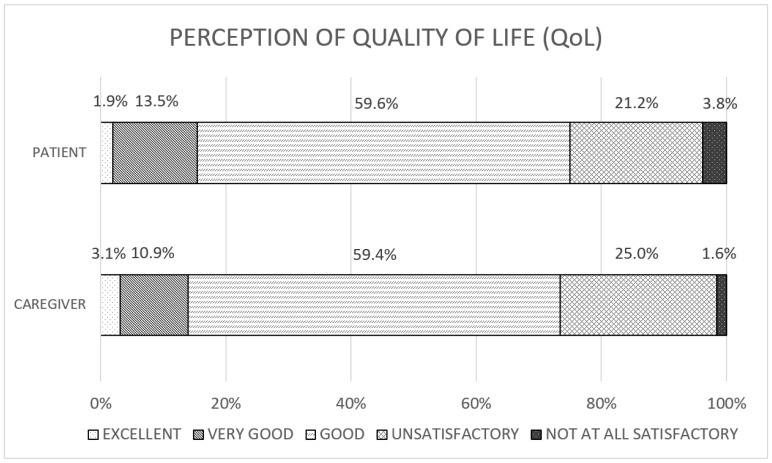
Perception of Quality of Life. Rows show the distribution of Quality of Life (QoL) perception, referred to as excellent (pinpoint dot on white background), very good (diagonal line), good (horizontal lines), unsatisfactory (checkered) and not at all satisfactory (pinpoint dot on black background). At the top, patients; below, caregivers.

Altogether, 75.0% of patients described their QoL as good at least; in particular, 1.92% described it as optimal, 13.46% as very good, and 59.62% as good (Figure 2).

Furthermore, 65.4% of patients declared that they had sufficient resources to deal with the situation. 

There was a weak correlation between QoL and resources in patients (ρ = 0.37, *p* ≤ 0.001).

Of the caregivers, 73.44% defined their QoL as good at least (3.13% as optimal, 10.94% as very good, and 59.38% as good) (Figure 2); only 22.73% of caregivers reported their care burden had increased during the pandemic, and the care burden did not relate to QoL. 

There was a weak correlation between patient and caregiver QoL (ρ = 0.31, *p* = 0.0154).

**Figure 3:**
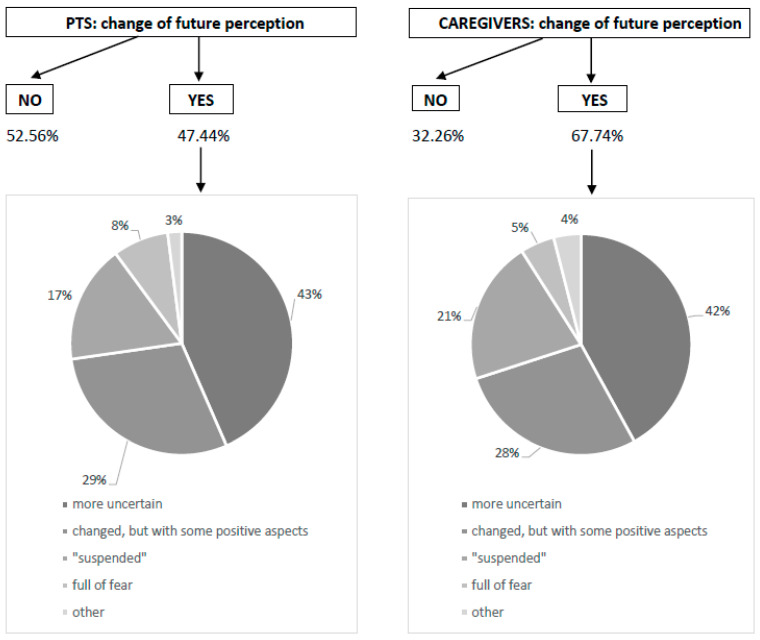
Change of future perception. The graph shows the distribution between the presence or not of modification of future perception, for patients and caregivers, respectively. The pie-chart reports the distribution among the different perception profiles, described as “more uncertain” (very dark gray), “changes, but with some positive aspects” (dark gray), “suspended” (gray), “full of fear” (light gray) and other (very light gray).

Altogether, 47.44% patients felt a different perception of the future during COVID-19, and now they feel a higher sense of uncertainty (43.02%), a sensation of a “different future” that they cannot further define (29.07%), a feeling of “suspension” (17.44%), fear (8.14%), or other (Figure 3). In 67.74% of caregivers, the perception of the future has been changed, mostly towards greater insecurity (41.86%) (Figure 3).

**Figure 4:**
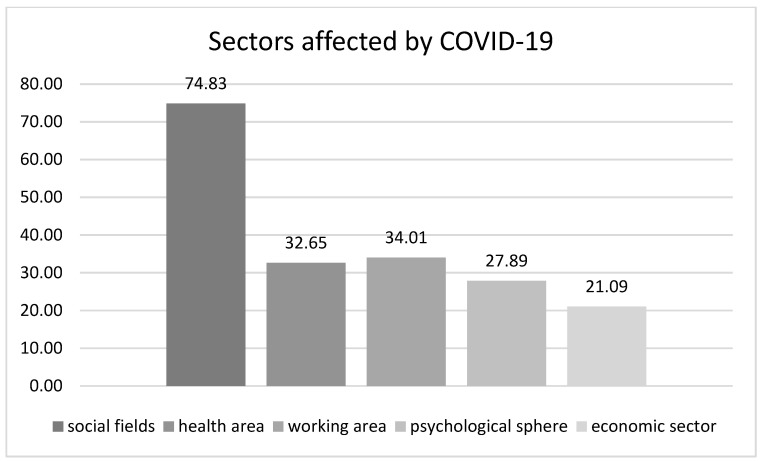
Sector affected by COVID-19. The graph shows the distribution of the sectors of human life affected by COVID-19 for patients. The pie-chart reports the distribution divided into social fields (very dark gray), health area (dark gray), working area (gray), psychological sphere (light gray), and economic sector (very light gray).

The COVID-19 pandemic influenced mostly the social fields (74.83%) and to a lesser extent the health and work area (32.65% and 34.01%, respectively), the psychological sphere (27.89%), and the economic sector (21.09%) for the patient cohort (Figure 4). Some patients experienced the impact in more than one sector.

**Figure 5:**
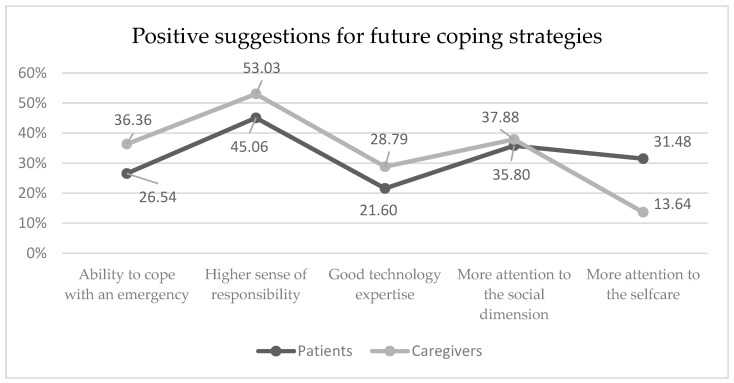
Positive suggestions for coping strategies. The graph shows the difference in the distribution of positive suggestions for improving coping strategies in the future for patients (very dark gray) and caregivers (dark gray).

However, the COVID-19-forced modification of everyday life could result in some positive suggestions for future coping strategies, such as the ability to cope with an emergency (26.54% and 36.36%, patients and caregivers, respectively), a higher sense of responsibility (45.06% and 53.03%), good technology expertise (21.60% and 28.79%), and more attention to the social dimension (35.80% and 37.88%) and to the care of self (31.48% and 13.64%) (Figure 5). To successfully deal with cancer anxiety, some anecdotal strategies reported in our cohort were meditation, mindfulness, having sex, psychiatric drugs, psychotherapy (individual or group), and prayer.

### 3.3. Relationship with Healthcare Personnel and Medical Institutions

Due to the COVID-19 pandemic, 9.15% of patients decided to delay their anti-tumoral therapeutic schedule, and 27.85% said that they were worried about going to hospital for a consultation. Accordingly, we found a significant association between patients’ spontaneous therapy delay and patient–doctor relationship modification (*p* = 0.022, Fisher’s exact test).

Overall, during the COVID-19 period, a larger number of patients experienced no changes in treatment timing (81.5%) or in patient–doctor relationships (81.0%); 93.1% of patients were satisfied with the treatment received. 

## 4. Discussion

As widely reported, the COVID-19 pandemic resulted in cancer care delay in a large range of cancer settings (an ASCO survey reported that half of patients with active cancer experienced a negative impact on their cancer care https://www.asco.org/research-data/reports-studies/national-cancer-opinion-survey, accessed on 10 June 2024); in the neuro-oncological setting the pandemic changed treatment schedules and limited investigational treatment options [14]. However, a mono-institutional study reported that cancer care delay did not impact the outcome [15]. In our study, most of the patients (81.5%) had experienced no changes in treatment timing or cancer care.

On the other hand, patients themselves could choose to defer oncological treatment based on their fear of SARS-CoV-2 exposure. We found that 9.15% of patients decided to delay their anti-tumoral therapeutic schedule, and 27.85% referred to being worried about going to hospital for a consultation. Furthermore, 9% of patients refrained from consulting a doctor or visiting the hospital due to fear of contracting the virus, according to Jeppesen (2021) [16]. COVID-19-related anxiety discouraged treatment in breast cancer patients [17]. To manage such issues, as well as to protect patients from SARS-CoV-2 exposure, we developed a telehealth intervention that provided a safe and easy way for patients to access their doctors, if applicable [18].

The emotional impact of COVID-19 is also measured by the perception of SARS-CoV-2 infection risk: the neuro-oncological patients described a higher risk than the general population in our study.

Although cancer patients are regarded as a highly vulnerable population to SARS-CoV-2 infection and the development of more severe COVID-19 symptoms with any type of cancer [19], in our cohort 9.26% of neuro-oncological patients caught the SARS-CoV-2 infection, and this percentage was similar to the general Italian population (10.6% from the COVID-19 pandemic onset to December 2021, from https://covid19.who.int/region/euro/country/it, accessed on 10 June 2024).

In our cases, anxiety related to the tumor was higher than COVID-19-related anxiety. Similar results were described by Binswanger, who, measuring with the distress thermometer, reported the highest score for disease-correlation versus COVID-19-relation in the neuro-oncological population [20].

Furthermore, we report that tumor-related anxiety correlates with COVID-19 anxiety, as described in a similar oncological context [21].

Examples of strategies adopted to deal with cancer anxiety that emerge from our study are meditation, mindfulness, having sex, psychiatric drugs, psychotherapy, and prayer. These are very different from the examples reported for dealing with COVID-19 anxiety: information, attention to the rules, isolation, and use of protective devices.

We reported that 47.44% patients felt a different perception of the future during COVID-19, describing a higher sense of uncertainty, the general perception of a “different future”, or a feeling of “suspension” or fear. 

A survey of 1079 patients with multiple myeloma showed that they had concerns about the future and events ahead, worries about family, friends, and relatives, and also have paternal irritation, feelings of sadness, anger, fear, and loneliness, and problems communicating with their spouses during the COVID-19 pandemic [22]. Moraliyage reported that the most important fears of the individual in the COVID-19 pandemic were fear of infection, weak immunity against the virus, travel, and caution among caregivers, as well as fear of supporting the family and others, fear of social isolation, and fear of infection [23]. Guven found more than 90% of cancer patients had moderate to severe fear of COVID-19 [24]. 

Besides the patient survey, we evaluated in parallel their caregivers. Caregivers are often overburdened with the situation of taking care of the patients, and increased risk for stress and mood impairment can even be associated with higher morbidity and mortality [25].

The origin of the stress, the goals, the appraisals, and the coping strategies of each individual and patient/caregiver dyads need to be considered to better manage the therapeutic path and to support families. One study reported that although the caregivers felt well supported by their social environment, stress, anxiety, and depression were common phenomena in neuro-oncology, especially for the female gender [26].

In our study patients’ and caregivers’ QoL are weakly correlated. Guariglia et al. (2021) [27] reported that HGG patients’ (*N* = 24) and caregivers’ perceptions of QoL were correlated between them and with the Karnofsky Performance Status (KPS). From a dyadic perspective, the adaptation of a member of the couple varies as a function of the other partner’s coping style. Others reported that QoL measures between patients and their families were weakly or moderately correlated [28].

As Baumstarck et al. (2018) [29] described, coping strategies implemented by the high-grade glioma patients (*N* = 38) and their caregivers influenced their own QoL and the QoL of their relatives.

An appropriate health-system organization and special attention to patient–doctor communication can make the difference to QoL and future perception.

Some limitations of our study are related to the type of questionnaires that explore the different issues in a semi-structured, self-administered survey at a single time point. Such structures exhibit the advantages of collecting fair answers (with no influence of interviewer/Caregiver opinion) and of being fast and easy to complete, while not being completely exhaustive in grasping the details of the item studied and lacking a longitudinal evaluation of a patient’s perception change. 

Another limitation could be represented by the “monocentric” approach. However, it could have a great impact on our way of organizing work in the team.

The merit of the present survey is the collection of exclusive primary neuro-oncological cases, although including very different types of brain tumors, with a different prognosis and at a different stage of the therapeutic course as well as of follow-up.

The majority of patients with malignant tumors are not necessarily hospitalized and not all have access to the psychological support which may help them to cope with their fears, worries, fatigue, and anger, particularly during the COVID-19 pandemic restrictions. To overcome feelings of isolation, depressive states, and insecurity about future perspectives, further supporting offers are needed. In this study, the topics of meaning in life, having authentic and long-lasting relationships, and mindful encounters with nature were important topics. These are the domains of psychotherapy and spiritual care. Spirituality, understood in this broader and open context, can be seen as an individual resource for a patient’s resilience, which is “maintaining self-esteem, providing a sense of meaning and purpose, giving emotional comfort, and providing a sense of hope” [30] in personal crisis management. Such spiritual care approaches [31] can be easily incorporated into a more comprehensive treatment and support of tumor patients, particularly in times of pandemic restrictions.

The collected data will be useful to develop and ameliorate coping strategies (maintaining social connection, redeploying previous coping strategies, engaging with spirituality, acceptance, self-distraction, taking action, and positive re-interpretation), as well as to modify the healthcare system.

Setting up mental health facilities to mitigate pandemic-induced psychological impacts of any future eventualities can be of merit.

We unexpectedly verified at least good QoL in most of the studied fragile population, represented by neuro-oncological patients during the COVID-19 pandemic; QoL is also sustained by adequate personal resources. However, anxiety rates ranged from 4.6 to 7.7; the higher score was related to neuro-oncological disease rather than COVID-19, and by caregivers rather than patients. The pandemic affected tumor monitoring/therapy only marginally in our context: patients experienced treatment delay in less than 20% of cases [32]; surprisingly, 10% of the patients decided to postpone therapies. Contextually, the doctor–patient interaction was reported as good and did not change over time.

The context of disease and pandemic contributed to the feeling of a more uncertain future, as patients and caregivers declared.

## 5. Conclusions

Based on the WHO biopsychosocial model of “health”, good medical disease management must include patients’ and their caregivers’ psychological needs, which can be bolstered also by a proactive support program. In our data, patients and caregivers were satisfied with the information on COVID-19 effects provided by their oncologists; in this regard, we must consider communication as a key tool to ensure the best possible care for the patients and their caregivers. Our data also outline that results regarding emotional status and disease burden in such patients should be taken into account for daily patient care management and could be useful for better defining a dedicated care pathway. It was more relevant in the neuro-oncological setting during the COVID-19 pandemic, but we need to improve the routine use of the anxiety and QoL scale scores as indicators of our clinical work. 

We will need to ensure that the patient–clinician relation is part of the care and that it reflects the style of relation chosen by the patients: in such a way, we will act as allies in the continuum of care.

## Figures and Tables

**Table 1:** Demographic features of patients (A), their caregivers (B), and COVID-19 information sources (C).

**(A) Patients: Demographic and Tumor Data**		
**Patients (*N* = 162)**		
**Variable**	**Responses**	***N* (%)**
Gender	Male/Female	88 (54.3)/74 (45.7)
Age (years)	18–25	3 (1.8)
	25–40	50 (30.9)
	40–54	55 (34)
	55–69	47 (29)
	≥70	7 (4.3)
Nationality	Italian/Others	156 (96.3)/6 (3.7)
Tumor type	Gliomas	70 (46)
	Meningioma	38 (25)
	Medulloblastoma	10 (6.6)
	Ependymoma	8 (5.3)
	Neurinoma	5 (1.2)
	PCNSL	1 (0.6)
	Other	19 (12.5)
Tumor diagnosis	<3 months	10 (6.2)
	3–6 months	23 (14.2)
	6–11 months	15 (9.3)
	1–5 years	47 (29)
	>5 years	67 (41.3)
**(B) Caregivers’ Demographic Profile**		
**Caregivers (*N* = 66)**		
**Variable**	**Responses**	***N* (%)**
Gender	Male/Female	27 (40.9)/39 (59.9)
Age (years)	18–25	0 (0)
	25–40	8 (12.5)
	40–54	21 (31.8)
	55–69	33 (50)
	≥70	3 (4.6)
Nationality	Italian/Others	64 (96.9)/2 (3.1)
Kinship patients/caregivers	Patients’ parents	8 (12.1)
	Husband/Spouse	44 (66.7)
	Partner	4 (6)
	Sibling	3 (4.5)
	Other relatives	5 (7.6)
	Friend	1 (1.5)
	Other	1 (1.5)
**(C) Source of Information about COVID-19 Obtained by Patients**		
**Sources**		***N* (%)**
General Practitioner		61 (37.9)
Specialist doctor		27 (16.7)
Media		116 (71.6)
Web		76 (46.9)
Relatives and friends		38 (23.4)
Percentage of satisfaction with the information received		117 (72.2)

## Data Availability

The data that support the findings of this study are available from the corresponding author upon reasonable request.

## Supplementary Materials

The following supporting information can be downloaded at: https://www.mdpi.com/article/10.3390/curroncol31070288/s1, Supplementary Materials includes the Survey for Patients (S1) and the Survey for Caregivers (S2).
