# Supplementary material for: Critical Issues for Patients and Caregivers in Neuro-Oncology during the COVID-19 Pandemic: What We Have Learnt from an Observational Study
Source: Curr Oncol. 2024 Jul 4;31(7):3895–907. doi: 10.3390/curroncol31070288 (PMC11275436; doi:10.3390/curroncol31070288)
Supplement: Supplementary file 1 [file curroncol-31-00288-s001.zip › curroncol-2963355-supplementary.pdf]

Title of the study

Observational study on COVID pandemic effect in neuro-oncological patients and their caregivers

Study Code: Neo-CO

vers 1.0, 04.05.2020

COVID-19 (CORONAVIRUS SARS-CoV-2) PANDEMIC ERA: THE EXPERIENCE OF NEURO-ONCOLOGICAL PATIENT AND HIS CAREGIVER

PATIENT INTERVIEW (schedula A) CODE_____________

DEMOGRAPHIC DATA:

1. **AGE**
   - 18 - 25
   - 25 – 40
   - 40 – 55
   - 55 – 70
   - Over

2. **SEX**
   - Male
   - Female

3. **NAZIONALITY**
   - Italian (if yes, specify the region) _______________________________
   - foreign (if yes, specificy) _______________________________________

CLINICAL DATA:

4. **DISEASE DIAGNOSIS**
   - glioma (including GBM)
   - meningioma
   - ependimoma
   - medulloblastoma
   - PCNSL
   - neurinoma
   - other (specificy)____

5. **SINCE THE DISEASE WAS DIAGNOSED?**
   - Less than 3 months

- 3-6 months
- 6-12 months
- More than 1 year
- More than 5 year

6. TRATTAMENTS ALREADY DONE:

- cerebral biopsy
- neurosurgery
- Radiotherapy
- Chemotherapy
- Immunotherapy
- rehabilitation/speech therapy
- therapy (specify) ____

7. ONGOING TREATMENTS:

- Radiotherapy
- Chemotherapy
- Immunotherapy
- rehabilitation/speech therapy
- therapy (specify) ____

8. ARE YOU ENROLLED IN A CLINICAL TRIAL?

- Yes (specify) ____
- No

9. ANY SIGNS OF THE DISEASE:

- Speech/comprehension impairment (e.s: aphasia)
- Sensitive abnormalities
- Cognitive (memory, attention, spatial- temporal orientation, etc.) deficit
- Motor Deficit
- seizures
- headache
- other (specify) ____
- No

COVID 19 RELATED DATA:

10. HOW IS YOUR PERCEPTION OF THE RISK OF CONTRACTING THE VIRUS Coronavirus SARS CoV-2?

- same risk of general population
- higher risk of general population
- lower risk than general population

11. HAVE YOU BEEN TESTED FOR Coronavirus SARS-CoV-2?

- yes
- No

12. HAVE YOU COMPLAINED ANY SYMPTOMS RELATED TO COVID-19?

- yes (specify) ____
- No

13. **HAVE YOU GOT Coronavirus SARS-CoV-2 INFECTION?**
- yes
- No

14. **DO YOU KNOW PEOPLE WHO GOT Coronavirus SARS-CoV-2 INFECTION?**
- yes (specify) ___________________________
- No

DATA RELATED TO DISCOMFORT LINKED TO THE PRESENT LIFESTYLE OR WAY /CONDITION OF LIFE?:

15. **HOW DO YOU FEEL IN THIS PERIOD?**
- I feel I have sufficient resources to deal with the situation
- I'm worried
- I feel I've exhausted my resources
- Other (specify)____

16. **HOW IT WOULD ASSESS ITS ANXIOUS STATE RELATED TO THE DIAGNOSIS OF BRAIN CANCER? ON A SCALE OF 1 TO 10 (where 1 indicates total absence and 10 the highest level of anxiety)**

1 2 3 4 5 6 7 8 9 10

17. **HAVE YOU ADOPTED ANY STRATEGIES TO COMBAT ANXIETY? IF YES, WHICH ONES?**

____

____

____

18. **HOW WOULD YOU ASSESS YOUR ANXIETY ABOUT THE PERCEIVED RISK OF CONTRACTING COVID-19? ON A SCALE OF 1 TO 10 (where 1 indicates total absence and 10 the highest level of anxiety)**

1 2 3 4 5 6 7 8 9 10

19. **HAVE YOU ADOPTED ANY STRATEGIES TO COMBAT SUCH ANXIETY (RELATED TO COVID)? IF YES, WHICH ONES?**

____

____

____

20. **ARE YOU AFRAID OF THE CURRENT HEALTH STATUS RELATED TO COVID-19 (Coronavirus)?**
- yes
- No

DATA RELATIVE TO OVERALL CARE/SUPPORT:

21. HAVE YOU EVER PLANNED (OR SOMEONE PROPOSED TO YOU) ANY TYPE OF PSYCOLOGICAL SUPPORT?

- Yes, I'm following a psychological therapy
- No

22. WHAT DO YOU THINK COULD HAVE HELPED YOU IN THIS PERIOD OF LIFE?

- support groups -si chiamano così?
- Support material
- social relationship
- Interaction with other professionists to get more information
- other (specify) ____

23. In this period of limitation of movement and social contacts, technology can play an important role in reducing the isolation? Which tool you use?

- Smartphone for voice call
- Smartphone for videocall
- Computer for hobbies
- Computer for information and education
- other (specify)____

DATA RELATED TO QUALITY INFORMATION OBTAINED ON COVID-19 (Coronavirus):

24. WHAT SOURCES OF INFORMATION DO YOU CONSULT OR CONSULT?

- General doctor
- specialist
- Tv and newspaper
- Internet
- Relatives/friends
- other (specify)____

25. ARE YOU SATISFY OF THE INFORMATION QUALITY?

- yes, because____
- No, because____

26. IF YOU REPLIED "NO" AT QUESTION 25, PLEASE SPECIFY WHICH INFORMATION YOU WILLING TO HAVE?

____

____

____

DATA RELATIVE TO TUMOR MANAGEMENT:

27. HAVE YOU POSTPONED ANY NEURO-ONCOLOGICAL THERAPY OPTION DUE TO COVID-19 PANDEMIC?

- Yes (specify)____
- No

28. **HAVE YOU OPTED TO MODIFY NEURO-ONCOLOGICAL THERAPY DUE TO COVID-19 EPIDEMIC?**

- Yes (specify)____
- No

29. **ARE YOU WORRIED ABOUT COVID-19 ANY TIME YOU ACCESS TO THE HOSPITAL FOR NEURO-ONCOLOGICAL DISEASE EVALUATION/THERAPY?**

- yes
- No

30. **IF YOU REPLIED "YES" TO THE QUESTION 29: ARE THERE ANY STRATEGIES THAT THE HEALTH PERSONNEL COULD BE ADOPT TO DICREASE YOUR DISCOMFORT?**

____

____

31. **DO YOU FEEL ANY CHANGE WITH YOUR HEALTH PERSONNAL DURING COVID-19 ERA?**

- YES, with any health professionist
- Only with some
- No

32. **ARE YOU SATISFIED ABOUT THENEURO-ONCOLOGICAL TREATMENT MANAGEMENT?**

- yes (specify)____
- No (specify) ____

33. **DO YOU HAVE ANY SUGGESTIONS TO IMPROVE THE QUALITY OF INTERACTION BETWEEN YOU AND THE HEALTH PROFESSIONIST STAFF?**

____

____

EVERYDAY LIFE:

34. **WITH WHOM DO YOU LIVE?**

- alone
- with relatives
- other______________________________

35. **ARE YOU PRESENTLY ISOLATED DUE TO COVID-19 (Coronavirus)?**

- Yes
- No

36. **IF YOU ARE A WORKER: DO YOU THINK THAT YOUR WORK WILL BE AT RISK BECAUSE OF COVID-19?**

- Yes
- No

37. **WHICH ARE THE ASPECTS OF YOUR LIFE THAT HAVE BEEN MORE INFLUENCED BY COVID-19 PANDEMIC?**

- health
- work
- Social
- Economic
- Psyhcological
- Other

38. **HOW IS YOUR QUALITY OF LIFE NOWDAYS?**

- Optimal
- Very good
- good
- unsatisfactory
- not at all satisfactory

THE FUTURE:

39. **SINCE COVID-19 PANDEMIC, DO YOU CHANGE YOUR PERCEPTION ABOUT THE FUTURE?**

- yes
- No

40. **IF YOU REPLIED "YES" TO QUESTION 39, HOW DO YOU PERCEIVE YOUR FUTURE:**

- More uncertain
- modified, including also postiive aspects
- "Suspended"
- Full of fair
- Other (specify)____
  ____

41. **ARE THERE ANYTHING IN THE PRESENT DAYLIFE (MODIFIED DUE TO COVID-19), THAT COULD BE USEFUL FOR THE FUTURE ALSO?**

- Capacity to mamange any emergency
- Higher sense of responsabiltiy
- Better use of techonologies
- Attention to social dimension
- to take care of myself
- other (specify) ____

Would you like to add other suggestions/comments?

____

____

____

Title of the study
**Observational study on COVID pandemic effect in neuro-oncological patients and their caregivers**

Study Code: Neo-CO

vers 1.0, 04.05.2020

COVID-19 (CORONAVIRUS SARS-CoV-2) PANDEMIC ERA: THE EXPERIENCE OF NEURO-ONCOLOGICAL PATIENT AND HIS CAREGIVER

CAREGIVER INTERVIEW (schedula B) **CODE____________**

DEMOGRAPHIC DATA:

1. AGE

- 18 - 25
- 25 – 40
- 40 – 55
- 55 – 70
- OVER

2. SEX

- Male
- Female
-

3. NAZIONALITY

- Italian (if yes, specify the region) ________________________________
- foreign (if yes, specificy) ____

4. WHICH RELATIONSHIP TO YOU HAVE WITH THE PATIEN?

- Patient’s parent
- Patient’s wife/husband
- Patient’s partner
- Patient’s siblin
- Other patient’s relative
- Patient’s friend
- other (specificy)____

COVID-19 RELATED DATA:

5. ARE YOU WORRIED TO CONTRACT THE VIRUS Coronavirus SARS CoV-2?

- o I'm very worried
- o I'm quite worried
- o L'm not worried
- o I already got it

6. WHAT CONCERNS SHOULD YOU ADDRESS AS A CAREGIVER OF A PATIENT DIAGNOSED WITH BRAIN CANCER AT A TIME WHEN Coronavirus SARS-CoV-2 INFECTION HAS SPREAD?

- o I'm worried that the patient could not access to the treatment/follow-up
- o I'm worried that the patient could get Coronavirus infection
- o I'm worried to access to the hospital
- o I am concerned about the burdens of the practical management of cancer disease in a difficult context such as that caused by COVID-19 (booking visits, travel, appointments, administration of therapies with attention to distance, masks, caution and attention)
- o I'm worried about my work/economic situation
- o I have no particular worries about covid-19 pandemic

7. As a result of the spread of Covid-19 (Coronavirus), has the perceived burden of the assistance you provide to the person you are dealing with increased?

- o No, nothing changed
- o Yes (please, add a description)____

DATA RELATED TO DISCOMFORT LINKED TO THE PRESENT LIFESTYLE OR WAY /CONDITION OF LIFE?:

8. HOW IT WOULD ASSESS ITS ANXIOUS STATE RELATED TO THE DIAGNOSIS OF BRAIN CANCER OF YOUR DEAR? ON A SCALE OF 1 TO 10 (where 1 indicates total absence and 10 the highest level of anxiety)

1 2 3 4 5 6 7 8 9 10

9. HAVE YOU ADOPTED ANY STRATEGIES TO COMBAT ANXIETY? IF YES, WHICH ONES?

____

____

____

10. HOW WOULD YOU ASSESS YOUR ANXIETY ABOUT THE PERCEIVED RISK OF CONTRACTING COVID-19? ON A SCALE OF 1 TO 10 (where 1 indicates total absence and 10 the highest level of anxiety)

1 2 3 4 5 6 7 8 9 10

11. HAVE YOU ADOPTED ANY STRATEGIES TO COMBAT SUCH ANXIETY (RELATED TO COVID)? IF YES, WHICH ONES?

____

____

____

12. WHICH AREAS HAVE BEEN MORE AFFECTED BY COVID-19 PANDEMIC?

- health
- work
- social
- economic
- psycological
- other____________________

DATA RELATIVE TO OVERALL CARE/SUPPORT:

13. HAVE YOU EVER PLANNED (OR SOMEONE PROPOSED TO YOU) ANY TYPE OF PSYCOLOGICAL SUPPORT?

- Yes, I'm following a psychological therapy
- No

14. WHAT DO YOU THINK COULD HAVE HELPED YOU IN THIS PERIOD OF LIFE?

- support groups
- Support material
- social relationship
- Interaction with other professionists to get more information
- other (specify) ____

15. In this period of limitation of movement and social contacts, technology can play an important role in reducing the isolation? Which tool do you use?

- Smartphone for voice call
- Smartphone for videocall
- Computer for hobbies
- Computer for information and education
- other (specify)____

16. HOW DO YOU EVALUATE YOUR QUALITY OF LIFE NOWDAYS?

- optimal
- very good
- good
- unsatisfactory
- not at all satisfactory

THE FUTURE:

17. SINCE COVID-19 PANDEMIC, DO YOU CHANGE YOUR PERCEPTION ABOUT THE FUTURE?

- yes
- No

18. IF YOU REPLIED "YES" TO QUESTION 17, HOW DO YOU PERCEIVE YOUR FUTURE:

- More uncertain

- modified, including also postiive aspects
- “Suspended”
- Full of fair
- Other (specify)____

19. ARE THERE ANYTHING IN THE PRESENT DAYLIFE (MODIFIED DUE TO COVID-19), THAT COULD BE USEFUL FOR THE FUTURE ALSO?

- Capacity to mamange any emergency
- Higher sense of responsabiltiy
- Better use of techonologies
- Attention to social dimension
- to take care of myself
- other (specify) ____________________________________

Would you like to add other suggestions/comments?

____
